# Evolution, adaptation, and new applications of the FODMAP diet

**DOI:** 10.1002/jgh3.13066

**Published:** 2024-05-20

**Authors:** Leigh O'Brien, Arezina Kasti, Emma P. Halmos, Caroline Tuck, Jane Varney

**Affiliations:** ^1^ Department of Medicine University of Otago Christchurch New Zealand; ^2^ Department of Nutrition and Dietetics ATTIKON University General Hospital Athens Greece; ^3^ Department of Gastroenterology Monash University and Alfred Health Melbourne Victoria Australia; ^4^ Department of Nursing and Allied Health Swinburne University Melbourne Victoria Australia

**Keywords:** irritable bowel syndrome, luminal gastroenterology, diet

## Abstract

The FODMAP diet has been a treatment of irritable bowel syndrome (IBS) for many years. Rigorous scientific evaluation and clinical application of the FODMAP diet have generated deep understanding regarding clinical efficacy, mechanisms of action, and potential adverse effects of this dietary approach. In turn, this knowledge has allowed fine‐tuning of the diet to optimize treatment benefits and minimize risks, in the form of the traditional three‐phase diet; the FODMAP‐gentle approach, which is a less restrictive iteration; and a proposed FODMAP‐modified, Mediterranean‐style diet which endeavours to optimise both gastrointestinal symptoms and other health parameters. Furthermore, recognition that IBS‐like symptoms feature in other conditions has seen the FODMAP diet tested in non‐IBS populations, including in older adults with diarrhea and women with endometriosis. These areas represent new frontiers for the FODMAP diet and a space to watch as future research evaluates the validity of these novel clinical applications.

## Graphical abstract

This paper summarizes the evolution of the FODMAP diet, and explores some of the newer applications and adaptations of this diet therapy, including a proposed five‐phase FODMAP diet framework.

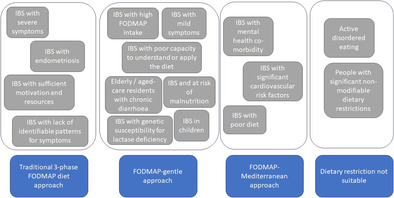

## Introduction

Supported by almost two decades of research, consistent evidence from randomized controlled trials (RCTs) and meta‐analyses has shown that a low‐FODMAP diet can reduce gastrointestinal (GI) symptoms, with effects on symptom response superior to other dietary interventions.^1^ Consequently, the FODMAP diet is now widely recommended as a first‐ or second‐line therapy in multiple clinical guidelines.^2^, ^3^, ^4^

The intensive interest and scientific appraisal of this diet has generated a depth of knowledge not only about clinical responses, but also about the FODMAP composition of food, mechanisms of action, and adverse effects, and it has allowed a fine‐tuning of the approach to optimize outcomes and mitigate adverse effects. Functional GI symptoms feature in numerous other conditions, and this has led to the exploration of the FODMAP diet in older adults with diarrhea,^5^ mothers of infants with colic,^6^ people with inflammatory bowel disease,^7^ people with functional dyspepsia,^8^ and women with endometriosis.^9^ This paper summarizes the evolution of the FODMAP diet, and explores some of the newer applications and adaptations of this diet therapy.

## Evolution of the traditional three‐phase FODMAP diet: Fine‐tuning FODMAP diet delivery

Since its inception and original publication in 2005,^10^ the FODMAP diet has evolved into what is now known as a three‐phase FODMAP diet.^11^ This treatment protocol describes: Phase 1, an initial restriction of foods known to contain high amounts of FODMAPs; then, if symptomatic response is achieved, progression to Phase 2, which is a series of strategic reintroductions of specific foods to assess tolerance to the various FODMAP types; and lastly, Phase 3, a personalized long‐term diet tailored to control irritable bowel syndrome (IBS) with minimal FODMAP restriction required. While the effect of individual FODMAP subgroups on symptom induction was long known,^12^ their combination under the umbrella term “FODMAP” revolutionized dietary therapies for IBS.

Mechanistic understanding has evolved from studies that originally described the osmotic actions in the small intestine via ileostomates^13^ and fermentation in the large intestine resulting in gas production shown in breath hydrogen.^14^ Findings from these mechanistic studies were confirmed by magnetic resonance imaging (MRI) studies, which highlighted the diverse effects of FODMAPs in the gut. For instance, excess fructose was shown to be more osmotically active than fructans, which exert effects more so via fermentation in the colon.^15^ Furthermore, an MRI study showed that whereas physiological responses to FODMAPs were similar between healthy subjects and people with IBS, symptoms were exaggerated in the IBS group, indicating that visceral hypersensitivity, not excessive gas production, provokes symptoms in IBS.^16^ Following this, a study using MRI of both the gut and brain showed that pain‐related brain regions responded to fructans differently in IBS patients compared to healthy subjects and that symptom induction coincided with colonic gas and excitation of pain‐related regions of the brain. This suggests that gut–brain axis dysregulation may drive FODMAP‐induced symptom generation in IBS.^17^ Additionally, a reduction in abdominal pain reported by patients on a low FODMAP diet was associated with reduced nociceptive signaling, as shown by afferent nerve recordings with fecal supernatants from a low‐FODMAP intervention, as compared with supernatants prior to dietary manipulation.^18^ The reverse translational experiments also suggested that histamine and proteases are likely to be involved in the pain response seen prior to dietary intervention, suggesting that modulation of these may represent an additional mechanism of action of the low‐FODMAP diet.^18^ Finally, intestinal permeability has been suggested to play a role, whereby reduced colonic epithelial injury occurred with lower FODMAP intake.^19^ Taken collectively, while acute effects such as reduced osmotic action and gas production clearly occur when FODMAP intake is reduced, emerging evidence suggests that reducing FODMAP intake alters other pathophysiological mechanisms that drive IBS, and this may allow for a more targeted application of the diet in the future.

Along with the growing understanding of FODMAP mechanisms and food composition,^20^ efficacy data developed. Studies showing symptom benefit have now been conducted across five continents.^21^ While adaptations are required, sufficient FODMAP reduction to achieve symptom response appears possible across cultures.^21^ However, as efficacy data strengthened for the restrictive phase, safety concerns emerged regarding the effect of this diet on nutritional adequacy, microbiome composition, quality of life (QOL), and disordered eating risk.^22^ These clinical concerns drove the development of re‐challenge protocols to determine specific FODMAP triggers and to allow for dietary liberalization.^23^ Ultimately, this led to the development of the three phases of the diet (including the restriction, re‐challenge, and long‐term phases).^11^ A limited number of studies assessing the re‐challenge^24^ and long‐term^25^ phases suggest that symptom response can be maintained despite FODMAP intake increasing toward quantities consumed at baseline. Dietetic support appears to improve adherence and enable patients to implement all three phases of the diet.^26^ While efficacy data are strong for the restrictive phase of the diet, prospective studies are needed to better understand the re‐challenge and long‐term phases.

**Figure 1:**
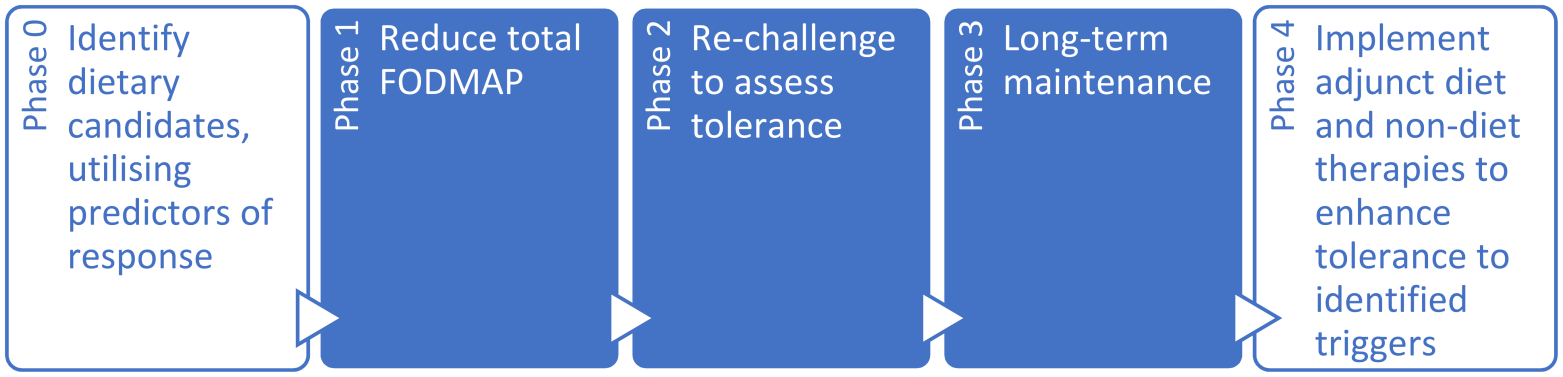
Proposed new five‐phase FODMAP diet framework. In this updated framework, the diet is a five‐phase approach including two newly described phases of predicting response to identify ideal candidates (Phase 0), and implementation of adjunct diet and non‐diet therapies (Phase 4).

While current practice is for the FODMAP diet to be followed in three phases, in the future, a five‐phase approach may prove both possible and optimal (Fig. 1). This would begin with predicting response to the diet and instituting the diet only in those most likely to benefit via fecal microbiota,^27^ volatile organic compound,^28^ symptom, and/or psychological profiling.^29^ This may be incorporated into clinical practice via fecal profiling or questionnaires measuring symptom and/or psychgolocial predictors, although until further supportive evidence becomes available, these are not ready for primtime in clinical practice. Following the three‐phase diet, an additional phase may be incorporated, whereby other diet and non‐diet adjunct therapies (such as targeted enzyme therapies for lactose^30^ and galacto‐oligosaccharides,^31^ and/or alterations to food processing or cooking^32^) could be used to optimize treatment response. This additional phase (Phase 4) may be incorporated alongside Phase 3 to maximize dietary variety and minimse symptom response where appropriate.

While the clinical efficacy and mechanisms of action of the FODMAP diet in IBS are now well established, the diet does have its drawbacks, making it unsuitable in some patient groups. For instance, effects on psychological symptoms appear mixed,^33^ and long‐term adherence to the restrictive phase of the diet may compromise nutritional intake and alter the gut microbiota.^34^ The diet is also restrictive and relatively complex, such that it is unsuitable in people with limited interest or skills in preparing food, those with a current or past history of disordered eating, people following an already restricted diet, people at nutritional risk, people who are underweight and/or experiencing unexplained weight loss, frail and older adults, and young children.^35^

**Figure 2:**
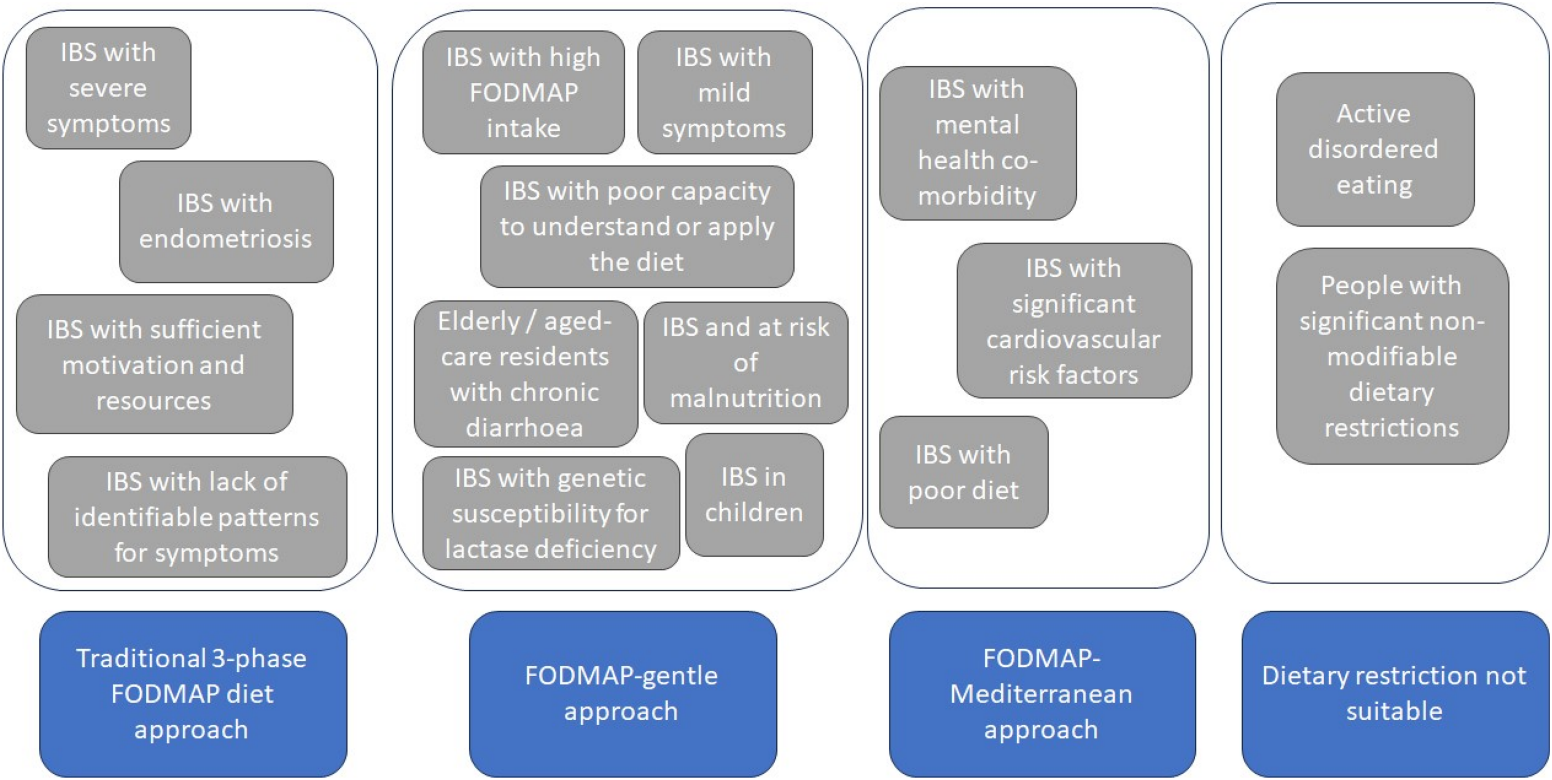
Adaptation of the FODMAP diet in people with irritable bowel syndrome (IBS)—indications for a traditional three‐phase FODMAP approach, a FODMAP‐gentle approach, a FODMAP–Mediterranean approach, and non‐dietary approaches.

To overcome these problems and meet the clinical needs of some of the above patient groups, the diet has undergone adaptations. These adaptations have seen the FODMAP diet simplified, with fewer dietary restrictions (in the form of the FODMAP‐gentle approach)^36^ and altered to incorporate features of the Mediterranean diet^37^ (Table 1, Fig. 2). Furthermore, recognition that functional GI symptoms are not unique to people with IBS has seen the FODMAP diet applied to new patient groups, including women with endometriosis^9^ and older adults with chronic diarrhea^5^ (Table 2).

**Table 1:** Adapting the FODMAP diet in irritable bowel syndrome (IBS) patient groups—ideal candidates and key considerations

	Ideal IBS candidates	Key considerations
Traditional three‐phase FODMAP approach	Patients who Have more severe symptoms Lack of identifiable patterns for symptom generation with food^38^ Have sufficient motivation and resources to conduct the diet in full	Current evidence best supports this approach Dietitian‐delivered education superior to booklet‐delivered education^39^ More restrictive dietary adjustments required at outset as compared to FODMAP‐gentle approach, but may be better able to identify specific food triggers and hence improve long‐term success of the diet^40^
FODMAP‐gentle approach	Patients who are Eating large concentrations of FODMAPs Mildly symptomatic Children Elderly Nutritionally compromised Following other dietary restrictions At poor capacity to understand and/or apply the diet Preferred to follow this approach	Dietetic assessment of habitual diet will guide the choice of diet application Robust trial evidence for the FODMAP‐gentle diet is lacking Traditional IBS dietary advice shares features of a FODMAP‐gentle diet, with some evidence of efficacy for symptom management
Low‐FODMAP Mediterranean diet	Psychological symptoms (anxiety/depression) Cardiovascular risk factors Patients with poor‐quality diets	Time to prepare meals Adequate financial resources Access to Mediterranean foods

**Table 2:** New applications of the FODMAP diet—ideal candidates and key considerations

Population	Ideal candidates	Key considerations
Inflammatory bowel disease	Patients with quiescent IBD and persistent functional gut symptoms (e.g. bloating, wind, abdominal pain)	Patients with IBD are susceptible to disordered eating practices and restrictive eating behavior is common in this population A limited trial of a FODMAP gentle diet can be considered, but dietetic guidance is recommended, and patients should be monitored for symptom response and impacts on food‐related quality of life
Older adults	Aged‐care residents who have been fully investigated for any non‐functional causes of diarrhea Those in whom a thorough medication review has been conducted to determine whether digestive symptoms such as diarrhea are not due to medications or laxative use^5^ Residents living in an aged‐care facility where the kitchen staff are able to accommodate dietary changes	Older adults have higher dietary protein and calcium requirements than younger adults, so careful consideration is needed when advising changes to lactose‐containing products^41^ Older adults prefer verbal communication A FODMAP‐gentle approach may be easier to implement in an aged‐care setting as meals are made in bulk Focus should be on substitution of high‐FODMAP foods to low‐FODMAP foods rather than elimination of food Good communication and detailed instructions are needed with the resident, care staff, GP, and kitchen staff for dietary changes to be implemented correctly
Endometriosis	Patients with endometriosis and co‐existing functional GI symptoms (e.g., abdominal pain, bloating, excessive wind, altered bowel habits). These symptoms may or may not meet Rome criteria for IBS.	Active screening for IBS in women with endometriosis and endometriosis with IBS is recommended (Table 4, ^9^) A limited trial of a three‐phase FODMAP diet can be considered, but given limited evidence for efficacy, patients should be supervised by a dietitian Symptom response before and after each phase of the diet should be monitored using a validated tool such as the VAS‐IBS^42^

GI, gastrointestinal; GP, general practitioner; IBD, inflammatory bowel disease; IBS, irritable bowel syndrome.

## Adaptation of the FODMAP diet: The “FODMAP‐gentle” diet

As with many therapies, clinicians have taken the liberty of adapting treatment protocols to meet the needs of individual patients. The FODMAP diet has been no different, with dietitians tailoring this diet therapy to meet the needs of patients in the form of the FODMAP‐gentle approach and the Mediterranean‐style FODMAP diet.

The ultimate aim of applying a FODMAP restriction is to find the minimal therapeutic dose for IBS management, thereby minimizing risks without compromising efficacy. One strategy of achieving this is the “top‐down” method of therapeutic dosing, as described through the aforementioned three‐phase FODMAP method of initial restriction of FODMAPs to assess response, reintroduction of FODMAPs, and then personalized long‐term FODMAP manipulation. However, “bottom‐up” strategies aimed to achieve the same outcome, coined “FODMAP‐gentle”, may also be applied.^36^ The concept of a FODMAP‐gentle diet is to only restrict a select few foods of very high concentration of FODMAPs and restrict further if adequate symptom control is not achieved. Table 3 describes a FODMAP‐gentle diet and suitable food alternatives.

**Table 3:** Description of a FODMAP‐gentle diet and food alternatives

Food group	Foods restricted on FODMAP‐gentle	FODMAP subgroup present	Low FODMAP food alternatives
Grains	Wheat‐ and rye‐based pasta, cereal, and bread	Oligosaccharides	Oat, rice, corn, gluten‐free pasta, cereal, and bread
Vegetables	Onion, leek, cauliflower, mushroom	Oligosaccharides, polyols	Chives, green part of spring onion, broccoli, green beans, pumpkin, capsicum, carrot, spinach
Fruit	Apple, pear, dried fruit, stone fruit, watermelon	Fructose, polyols, oligosaccharides	Banana, kiwifruit, blueberries, citrus fruits
Dairy	Cow's milk and yoghurt	Lactose	Lactose‐free milk and yoghurt, cheese, soy milk
Meat/alternatives	Legumes	Oligosaccharides	Tofu, eggs, meat, poultry, fish

To date, there are no trials evaluating the FODMAP‐gentle diet in treating IBS, but indirectly there are indications of its value beyond anecdotal experience. Prior to development of the low‐FODMAP diet, IBS management guidelines would recommend “traditional dietary advice” which was based largely on historical experience rather than trials.^43^, ^44^ Traditional dietary advice for IBS was to encourage a regular meal pattern; avoid excessively large meals; reduce fat, excessive fibre and caffeine intake; and reduce intake of gas producing foods, such as onions, beans and cabbage. This advice would incidentally reduce intake of many FODMAP‐rich foods, as recommendations would be to avoid wheat bread, wheat bran, legumes, onion, and soft drinks containing high‐fructose corn syrup.^45^ Additionally, these guidelines gave specific reference to avoiding particular FODMAPs such as lactose, sorbitol, and other polyols used as low‐joule sweeteners. This advice mirrors that of a FODMAP‐gentle diet. Thus, it may be extrapolated that the efficacy of traditional dietary advice for the treatment of IBS may, at least in part, be due to a reduction in FODMAP intake, as seen with a FODMAP‐gentle approach. Evidence for traditional dietary advice treating IBS is modest in comparison to a low‐FODMAP diet, with a meta‐analysis comparing the two dietary strategies finding that the low‐FODMAP diet is superior to traditional dietary advice for improving symptoms of IBS.^46^ However, data extracted from four RCTs assessing the traditional dietary advice still saw a statistically and clinically significant improvement in patients receiving traditional dietary advice, based on validated IBS scores.^46^ Controlled trial evidence evaluating a FODMAP‐gentle diet for treating IBS is much needed to substantiate such interpretation, particularly in an IBS population, with typically high placebo response.

Choosing the best FODMAP application is dependent on the patient assessment conducted by a dietitian.^47^ The benefit of the three‐phase FODMAP method is that it provides a clarity of response and as such is likely the best option for the majority of IBS sufferers, particularly where the success of the therapy is uncertain. While dietetic guidance does appear to negate the risk of nutritional inadequacy using a three‐phase FODMAP diet (likely due to careful assessment and monitoring),^21^ there are some populations that may benefit from a FODMAP‐gentle approach instead (Table 1, Fig. 2). For example,

- in patients who are mildly symptomatic or who include an excessive intake of FODMAPs, usually identified as part of a dietitian's assessment (e.g., patients consuming large volumes of apple juice);
- in vulnerable populations, particularly where diet quality or nutritional adequacy is compromised (e.g., other dietary restrictions in place such as a vegan or vegetarian diet, other comorbidities that increase nutritional risk such as inflammatory bowel disease);^36^
- at certain life stages (e.g., in children, where growth and development is a priority; pregnancy and breastfeeding; and adolescence where eating behavior is important and restrictive eating may increase risk of developing eating disorders);^21^, ^48^, ^49^
- in genetically susceptible individuals (e.g., lactose is more commonly poorly absorbed in those of East Asian decent);^38^ and
- in patients with limited capacity to understand and/or apply the diet (e.g., people with limited financial resources and older adults).

## Adaptation of the FODMAP diet: Mediterranean‐style FODMAP diet

So too, the FODMAP diet has undergone adaptation to reflect the eating style of a Mediterranean diet. The Mediterranean diet is a cultural heritage in the Mediterranean basin. This geographic area spans 20 countries across Europe, Asia, and Africa,^50^, ^51^ and despite some dietary variation between regions, there are numerous common features. The Mediterranean diet is rich in plant foods such as wholegrain sourdough bread and cereals, seasonal vegetables and fruits, legumes, nuts, and seeds. Extra virgin olive oil is used as the main source of added fat, dairy is included daily but from non‐sweetened, fermented sources such as yoghurt and feta cheese, and white meats such as poultry and fish are eaten in preference to red and processed meats, which are limited. Several servings of legumes and lentils are included each week, as are free‐range eggs. Herbs, spices, and lemon juice are used to flavor foods, ultra‐processed foods are limited, water is the main drink, and up to one glass of red wine is consumed daily, but always with meals.^52^

The Mediterranean diet is one of the most studied dietary patterns worldwide, with strong evidence from RCTs and meta‐analyses indicating that the diet reduces the risk of developing various chronic diseases (notably cardiovascular disease, cancer) and improves longevity.^53^, ^54^ Among the wide‐ranging effects of this dietary pattern, numerous studies have highlighted the anti‐inflammatory properties of the diet and shown that adherence alleviates symptoms of depression and anxiety^55^, ^56^, ^57^ and leads to favorable changes in the gut microbiota.^58^, ^59^ These observations have led some researchers to investigate the efficacy of a Mediterranean diet in the management of IBS, given the roles that an altered microbiome, low‐grade inflammation, and psychological disturbance play in the pathophysiology of this condition.

The concept of the combined FODMAP and Mediterranean diet started from data showing that low adherence to the Mediterranean diet was associated with a higher prevalence of IBS. This cross‐sectional study showed that a standard Mediterranean diet was not associated with disease severity, whereas certain Mediterranean foods were associated with symptoms and may not be suitable for all IBS patients, and rather that personalized management was needed for people with severe symptoms.^60^ Following this observation, a Mediterranean diet was compared with a low‐FODMAP diet and a gluten‐free diet, to evaluate feasibility via measures of acceptance and adherence, QOL, and GI symptoms. All three diets improved IBS symptoms and QOL but participants expressed a preference for the Mediterranean diet, likely due to ease of implementation.^61^ A recent unblinded RCT conducted in participants with IBS and mild or moderate anxiety and/or depressive symptoms randomized 59 participants to Mediterranean diet advice from a dietitian or habitual diet. The study showed that the Mediterranean diet (which was not low in FODMAPs) improved GI symptom severity, depressive symptoms, and QOL, with no changes in microbiome diversity, relative abundance, or functional potential in either group.^37^ These studies suggest potential for the Mediterranean diet in IBS, despite the diet including numerous high‐FODMAP foods, such as legumes. Novel approaches would be to compare benefits of a traditional Mediterranean diet with a low FODMAP‐Mediterranean diet to assess whether this can harness the benefits of both diets. While no data are yet available, the diet is currently being compared to nutritional recommendations for IBS from the British National Institute for Health and Care Excellence (NICE).^62^ Until more data become available for this novel approach for GI symptoms, current practices may consider using a Mediterranean diet instead of a low FODMAP diet where other co‐morbidities with known benefits from the Mediterranean diet are present, such as anxiety or cardiovascular risk factors.

## New applications of the FODMAP diet: patients with inflammatory bowel disease

IBS‐like symptoms are common in other populations, including those with another primary diagnosis. Functional gut symptoms are often out of step with identified active disease seen in IBD, with up to one‐third of those with IBD experiencing IBS‐like symptoms,^63^ although there are limitations in effectively determining true remission in IBD. A low FODMAP diet has been investigated in many studies, including well‐controlled trials of patients with quiescent IBD and IBS‐like symptoms,^64^, ^65^ and a meta‐analysis indicating that a low FODMAP diet improves overall gut symptoms above comparative diets.^66^ IBD patients may be considered a vulerable population in regards to dietary burden, as often they may have other dietary restrictions and some evidence suggesting that IBD patients are at higher risk of disordered eating behaviour, although eating disorder screening tools have not been validated in this population.^67^

## New applications of the FODMAP diet: Older adults with chronic diarrhea

Just as the FODMAP diet has undergone adaptation in the form of the FODMAP‐gentle and Mediterranean‐style aproaches, it has also been applied to newer populations, including older adults with chronic diarrhea and people with endometriosis. The efficacy of the low FODMAP diet has primarily been tested in adults younger than 65 years. However, older adults have specific dietary needs (such as higher requirements for calcium and protein) and many older adults to not meet the recommended dietary intake (RDIs) for these important nutrients on their habitual diet.^68^ Therefore, the effectiveness and safety of restrictive diets in this population needs careful consideration.

Chronic diarrhea is debilitating for older adults, with negative impacts on social interactions and QOL. Although prevalence data and understanding of this condition is limited, a study of GI symptoms and FODMAP intake among 74 residents of a New Zealand (NZ) aged‐care facility showed that half had functional GI symptoms. It also showed that 20% (*n* = 15) had a diagnosis of a lower GI disorder, with 8 residents experiencing chronic diarrhea (11%). Discomfort due to diarrhea was experienced by 39% of residents (*n*= 30), with 10 residents (13%) reporting diarrhea to be moderate to severe.^69^ Interestingly, laxative use was common among residents, including those with diarrhea (*n* = 69, 93%).

This observation regarding laxative use has been reported elsewhere. For instance, a recent retrospective observational study that collected data from aged‐care residents (*n* = 2411) living in 36 NZ aged‐care villages found that 65% of residents experiencing chronic diarrhea were taking laxatives at the time diarrhea occurred (*n* = 404).^70^ As such, it is recommended that medication charts be checked for laxatives when diarrhea is present. In addition, dietary fiber intake should be reviewed, given the evidence that provision and intake of dietary fiber is often inadequate among aged‐care residents.^71^ Addressing any fiber deficit via a food‐first approach may reduce the need for laxatives to treat constipation, thus preventing the unwanted consequences of diarrhea.^68^

A low‐FODMAP diet has emerged as an effective therapy to manage chronic diarrhea in older adults. This was highlighted in an unblinded observational pilot study of 20 participants (mean age 76 years) conducted in NZ community‐living older adults with chronic diarrhea,^5^ all of whom had undergone investigation for unexplained diarrhea. Following 6 weeks on the restrictive phase of the low‐FODMAP diet taught and supervised by an NZ‐registered dietitian, there was an improvement in GI symptoms, with 90% experiencing improvement in diarrhea/incontinence‐specific symptoms. Furthermore, 70% experienced fewer loose bowel motions. While the mean FODMAP intake reduced, there were no changes in intake of energy, protein, or fiber over the 6‐week intervention. While 40% of participants did not meet their RDI for calcium after diet, this was true of their intake before diet. All 19 participants who provided feedback on dietary acceptability reported that the diet was taught in a way they could easily understand. However, 57% of participants reported that they would not be able to follow the diet correctly if instructions were provided only in a written format (without any verbal instruction from the dietitian). These findings indicate that a low‐FODMAP diet could be considered for older adults living with chronic diarrhea in the community.

If diarrhea is considered to be functional, and unrelated to laxative use, a FODMAP‐gentle approach could be suitable in older adults, who are often over‐treated medically and are at risk of being over‐treated through diet too.^72^ Given FODMAP estimations of supplied food in nursing homes which reveal that fruit and dairy (particularly milk, pears, and prunes) contribute to the majority of FODMAP intake,^64^ simple menu changes toward a FODMAP‐gentle diet could be easily achieved, without compromising nutritional quality.^69^, ^72^ Of course, clinicians should account for the specific dietary requirements of older adults and the nutritional adequacy of the habitual diet.^73^ Furthermore, an experienced dietitian should provide individualized instruction and monitoring, given the increased nutritional requirements of older adults and the complexity of the FODMAP diet. Importantly, generic diet sheets are unlikely to meet the needs of this age group.^21^ Also, it is recommended that all those involved in the person's care should be informed of dietary changes, including the care team (registered nurse, care staff, and general practitioner) as well as the kitchen staff (chef, cooks, serving staff) and next of kin, to ensure safe and effective implementation of the diet. Further research that better characterizes chronic diarrhea among older adults and assesses the efficacy and safety of dietary therapies is eagerly awaited.

## FODMAPs for endometriosis: A new target

Another newer application of the FODMAP diet is among people with endometriosis. Endometriosis is a chronic, inflammatory gynecological disease characterized by the presence of endometrial tissue outside the uterus.^74^ In Australia, the condition affects around 1 in 7 (14%) by age 44–49.^75^ but prevalence is higher in women with pelvic pain and/or infertility, affecting 30–50% of patients.^76^ Endometriosis is associated with a range of painful symptoms including chronic pelvic pain, dysmenorrhea (painful periods), dyspareunia (painful sex), dyschezia (painful defecation), dysuria (painful urination), fatigue, depression, and infertility.^77^ Symptoms range in severity from mild to debilitating, and can substantially compromise mental health and QOL.^78^

Among the symptoms that characterize endometriosis, functional GI symptoms are extremely common, affecting 75–98% of sufferers.^79^, ^80^, ^81^ These symptoms overlap with numerous GI disorders, most notably IBS. Indeed, in women with endometriosis the risk of IBS is 2–3‐fold greater than in healthy controls.^82^ The converse is also true, as women with IBS are thought to have a threefold risk of developing endometriosis.^83^ This overlap confuses the diagnosis of these two conditions, leading to issues of misdiagnosis,^81^ a problem compounded by the inability of many women with endometriosis to reliably distinguish GI from gynecological pain.^84^

Despite the high prevalence of GI symptoms in endometriosis, few treatments specifically target these symptoms, and some (such as progesterone and opioids) can make them worse.^85^, ^86^ Instead, endometriosis treatments tend to be surgical or pharmacological and associated with numerous downsides, such as ongoing endometriosis‐related symptoms, high out‐of‐pocket costs, bothersome side effect profiles, and high recurrence rates (40–50% 5 years after surgery).^87^ Not surprisingly, treatment dissatisfaction rates are high,^88^ and many women with endometriosis use self‐management strategies such as diet to manage their symptoms. For instance, cross‐sectional data from Australia showed that 76% of patients use self‐management strategies, and of these, 44% used dietary modifications. The majority of diets were restrictive in nature, most commonly involving the exclusion of gluten, FODMAPs, or dairy.^89^ Similarly, a U.K. survey of dietitians working with endometriosis patients (*n* = 21) and women with endometriosis (*n* = 1385) showed that all dietitians used the low‐FODMAP diet, and with modest success. Among patients, over 75% reported inadequately controlled GI symptoms, and the majority had tried dietary restrictions to manage these.^90^

Despite this uptake of diet therapies for endometriosis, limited data support the efficacy of any given dietary approach. This lack of evidence is reflected in clinical guidelines. While some guidelines recommend including a dietitian in the interdisciplinary care team of patients with endometriosis,^91^ most fail to mention diet therapies, or cite the lack of evidence supporting specific approaches.^92^ Instead, most studies in women with endometriosis have examined the efficacy of dietary supplements, not whole dietary approaches. While some have looked at the efficacy of a Mediterranean diet, the role of diet quality, and diets that restrict intake of components such as nickel, FODMAPs, gluten, and soy, few used an RCT design or measured the effect on GI symptoms.^93^, ^94^, ^95^

This lack of evidence contrasts the strong evidence base that now underpins the efficacy of a low‐FODMAP diet for IBS.^1^ Given the overlapping symptoms between endometriosis and IBS, the high rates of IBS in endometriosis, and the common pathophysiological factors driving the two conditions, a low‐FODMAP diet may yield similar improvements in this population. Two studies have investigated the effect of a low‐FODMAP diet for endometriosis. The first was a retrospective observational study in women with endometriosis and IBS (*n* = 59), which showed at least a halving of GI symptoms after 4 weeks on a low‐FODMAP diet.^9^ Another nonrandomized, nonblinded prospective study in 62 women with endometriosis found that adherence to either a low‐FODMAP diet or the “endometriosis diet” (which restricted red meat, gluten, dairy foods, sugars and sweeteners, soy products, and caffeine) for 6 months reduced pain and bloating and improved QOL. However, because data concerning the efficacy of the two dietary interventions were pooled, the independent effect of either was not reported.^96^

In summary, patients with endometriosis frequently present with GI symptoms and many meet the Rome IV criteria for IBS. This overlap highlights the need for clinicians to actively screen for GI symptoms in women with endometriosis, and endometriosis in patients with IBS (Table 4).^97^ In patients with diagnosed endometriosis, a dietary approach can be considered, keeping in mind that the quality of evidence supporting any given dietary approach is generally poor and further high‐quality research is required. Nonetheless, a low‐FODMAP diet does show promise, and in patients with accompanying GI symptoms with or without an IBS diagnosis, a limited trial of a three‐phase FODMAP diet can be considered, under dietetic guidance.

**Table 4:** Red‐flag symptoms of endometriosis^†^

Red‐flag symptom	Take particular note of
Family history of endometriosis	Especially in first degree relatives
Chronic pelvic pain	With or without cyclic flares
Dysmenorrhea	Affecting daily activities and QOL
Cyclical GI symptoms	Especially dyschezia
Cyclic urinary symptoms	Especially hematuria or dysuria
Infertility	Associated with one or more of the above symptoms or signs
Deep dyspareunia	May be described as sharp, stabbing or cramping

^†^ Endometriosis should be considered when one or more of the following signs and symptoms is present in women (including girls aged ≤17 years).^42^

GI, gastrointestinal; QOL, quality of life.

## Conclusion

Growing evidence supporting the efficacy of the FODMAP diet for IBS has driven widespread clinical uptake and innovations, both in the structure of the diet and the clinical application. So long as research can keep pace, these developments should pave the way for better clinical outcomes for people with IBS, as well as new treatment options for other conditions.
